# High-Throughput Assay and Discovery of Small Molecules that Interrupt Malaria Transmission

**DOI:** 10.1016/j.chom.2015.12.001

**Published:** 2016-01-13

**Authors:** David M. Plouffe, Melanie Wree, Alan Y. Du, Stephan Meister, Fengwu Li, Kailash Patra, Aristea Lubar, Shinji L. Okitsu, Erika L. Flannery, Nobutaka Kato, Olga Tanaseichuk, Eamon Comer, Bin Zhou, Kelli Kuhen, Yingyao Zhou, Didier Leroy, Stuart L. Schreiber, Christina A. Scherer, Joseph Vinetz, Elizabeth A. Winzeler

**Affiliations:** 1 The Genomics Institute of the Novartis Research Foundation, 10675 John J. Hopkins Drive, San Diego, CA 92121, USA; 2 Division of Pharmacology and Drug Discovery, Department of Pediatrics, University of California San Diego School of Medicine, 9500 Gilman Drive, La Jolla, CA 92093, USA; 3 Division of Infectious Disease, Department of Medicine, University of California San Diego School of Medicine, 9500 Gilman Drive, La Jolla, CA 92093, USA; 4 Broad Institute, 415 Main Street, Cambridge MA 02142; 5 Medicines for Malaria Venture (MMV), PO Box 1826, 20 Route de Pré-Bois, 1215 Geneva 15, Switzerland; 6 Department of Chemistry and Chemical Biology, Harvard University, 7 Cambridge Center, Cambridge, MA 02142, USA

**Keywords:** transmission, malaria, chemotherapy, gametocytes, *Plasmodium*

## Abstract

Preventing transmission is an important element of malaria control. However, most of the current available methods to assay for malaria transmission blocking are relatively low throughput and cannot be applied to large chemical libraries. We have developed a high-throughput and cost-effective assay, the Saponin-lysis Sexual Stage Assay (SaLSSA), for identifying small molecules with transmission-blocking capacity. SaLSSA analysis of 13,983 unique compounds uncovered that >90% of well-characterized antimalarials, including endoperoxides and 4-aminoquinolines, as well as compounds active against asexual blood stages, lost most of their killing activity when parasites developed into metabolically quiescent stage V gametocytes. On the other hand, we identified compounds with consistent low nanomolar transmission-blocking activity, some of which showed cross-reactivity against asexual blood and liver stages. The data clearly emphasize substantial physiological differences between sexual and asexual parasites and provide a tool and starting points for the discovery and development of transmission-blocking drugs.

## Highlights

• Developed SaLSSA, a serum-free one-step assay for malaria transmission-blocking activity
• 13,983 known and new compounds analyzed by SaLSSA
• >90% known antimalarial drugs do not show activity against late-stage gametocytes
• Compounds with consistent low nanomolar transmission-blocking activity identified

## Graphical Abstract

**Figure:**
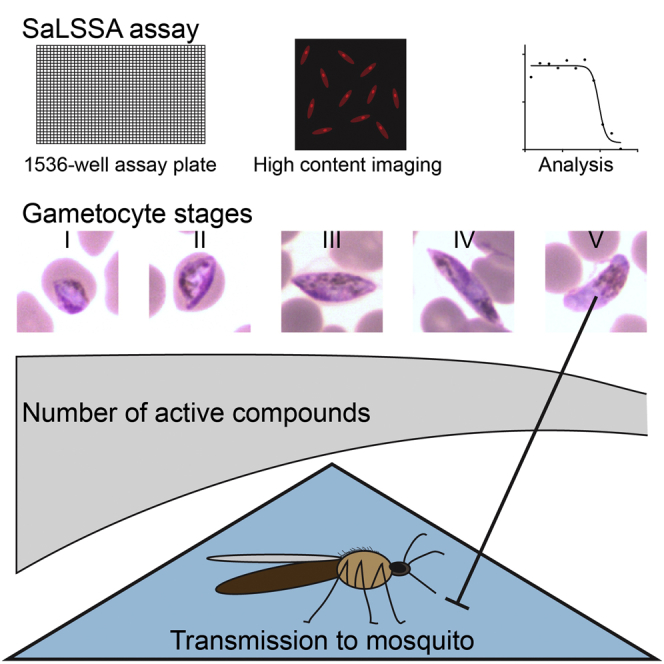

## Teaser

Preventing human-mosquito transmission is important for malaria control. Plouffe et al. developed SaLSSA, a one-step high-throughput assay to screen for malaria transmission-blocking activity. A large panel of known and new small molecules was analyzed by SaLSSA. This provides starting points for the discovery and development of transmission-blocking drugs.

## Introduction

Malaria is a vector-borne disease caused by apicomplexan eukaryotic protozoa of the genus *Plasmodium*. The parasites have a complex life cycle involving vertebrates and anopheline mosquitoes. Their asexual replication and destruction of erythrocytes give rise to the symptoms of malaria, including fever and chills. In response to cues that are not well understood, a subset of asexual parasites differentiate into male and female gametocytes (Sinden, 1983), a process that takes ∼8–12 days in *P. falciparum* (Sinden, 2009). During this period, the parasites metabolize the host red cell hemoglobin (Hanssen et al., 2012), while progressing through five morphologically distinct stages that can be identified by light microscopy (Carter and Miller, 1979). Commitment to sexual development occurs well before parasites show morphological changes, and male and female gametocytes are produced at a ratio of 1 to 3–5 (Gbotosho et al., 2011, Robert et al., 2003) with females maturing slightly later (Bounkeua et al., 2010). In the human body, immature gametocytes sequester in different host tissues (Rogers et al., 2000) and emerge only when fully mature. An infected individual may carry gametocytes for up to 55 days (Bousema et al., 2010), and mature gametocytes are the only form that can survive in the mosquito midgut, mate, undergo meiosis, and give rise to the next generation of parasites to be transmitted to a new human host.

Current first-line treatment of *falciparum* malaria is artemisinin combination therapies (ACTs) (WHO, 2015), which do not block transmission. Follow-up treatment with 8-aminoquinolines like primaquine or tafenoquine is needed to block transmission (Eziefula et al., 2014). However, 8-aminoquinolines can be toxic to individuals with glucose-6-phosphate dehydrogenase deficiency, a genetic condition with a high prevalence in malaria-endemic regions (Luzzatto, 1979).

Even though assays are available to detect compounds with transmission-blocking potential (Adjalley et al., 2011, Almela et al., 2015, D’Alessandro et al., 2013, Delves et al., 2012b, Duffy and Avery, 2012, Lelièvre et al., 2012, Lucantoni et al., 2013, Miguel-Blanco et al., 2015, Ruecker et al., 2014, Sun et al., 2014, Tanaka et al., 2013), most of them are not adapted for very large chemical libraries due to multiple purification steps or lower throughput formats. In addition, some assays rely on the use of gametocyte reporters that may restrict their use to genetically modified parasites (Adjalley et al., 2011, Peatey et al., 2011). Here we describe high-throughput assays that overcome these issues. We apply the assays to characterized and uncharacterized chemical libraries. Our analysis reveals features of chemical compounds that are likely to block malaria transmission and may serve as starting points for unique transmission-blocking drugs.

## Results

### Developing an Assay to Identify Compounds with Transmission-Blocking Activity

#### Production of Homogeneous Populations of Gametocytes

**Figure 1:**
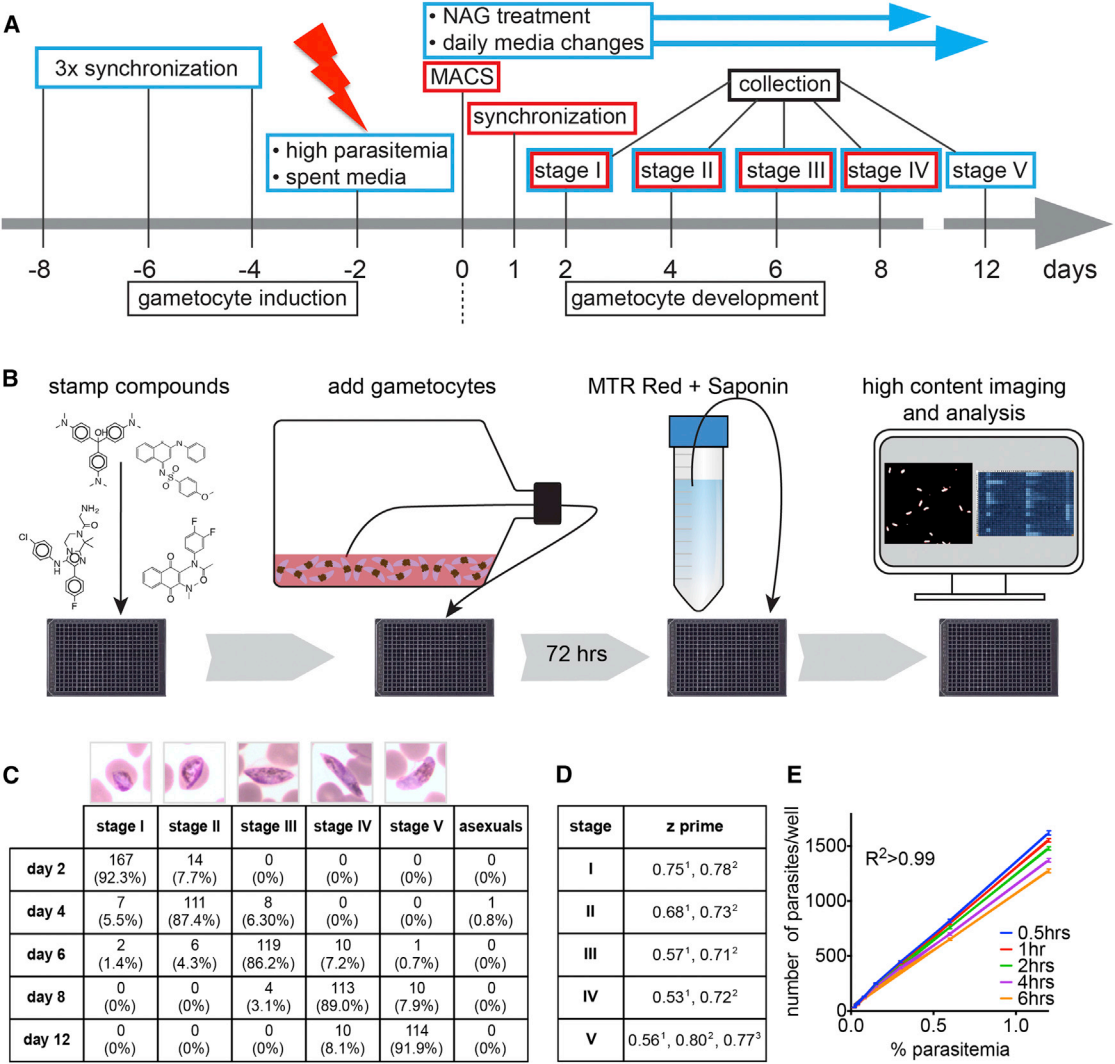
Induction and Development of Pure, Stage-Specific Gametocytes (A) Gametocyte production protocol. To create stages I–IV, all steps were performed, but to create stage V only, steps indicated by red boxes were omitted. (B) Simplified schematic flow chart for the SaLSSA gametocyte assay. (C) Giemsa smears of gametocytes of stage I-V and gametocyte counts and percentages for each stage based on their morphology (numbers pooled from multiple cycles). (D) *Z* prime values for the different gametocyte stages and assay protocols. ^1^384-well TSSA, stages I-V, ^2^384-well-SaLSSA, stages I-V, ^3^1,536-well SaLSSA, stage V. (E) Displayed is the number of viable stage V gametocytes in a dilution series over time (0.5 hr until 6 hr). These data were acquired using stage V gametocytes and 1,536-well SaLSSA (R^2^ > 0.99).

To create a homogeneous, stage-specific gametocyte population, we optimized a previously described protocol (Fivelman et al., 2007) and induced gametocytogenesis in asexual, triple synchronized *P. falciparum* NF54 parasites by high parasitemia and partly spent media (Figure 1A; Experimental Procedures). Microscopic staging of gametocytes collected over the 12 days of development according to description by Carter and Miller (1979) showed purities upward of 75% per stage (Figure 1C) with a reproducible parasitemia of 1.2%–1.6% over the screening period (data not shown).

#### Measuring Viability in Non-Replicating Parasites

To detect viability, we used the dye MitoTracker Red CMXRos (MTR Red), which fluoresces at ∼600 nm in parasites with intact mitochondrial membrane potential (Pendergrass et al., 2004, Poot et al., 1996) (Figure 1B). Parasites were detected using automated microscopy and showed a good correlation (R^2^ = 0.99) between the number of viable parasites added per well and the number of MitoTracker Red CMXRos positive objects (Figure 1E).

#### Reduction in Number of Liquid-Transfer Steps

To reduce the number of liquid transfer steps and make the assay more robust and less costly for use with large, unbiased libraries, we experimented with the use of saponin, an amphipathic glycoside that creates pores in red cell membrane bilayers, leading to red cell lysis (Baumann et al., 2000). We found that treating gametocyte cultures with 0.13% saponin caused red blood cells in serum-free media to lyse, simplifying the identification of parasites with automated microscopy. Gametocytes at a parasitemia of 0.5% to 0.75% and a hematocrit of 1.25% created a monolayer on the bottom of the well. After MTR Red staining ∼1,000 objects could be counted per DMSO control well (1,536) (Figure 1E). This allowed compound exposure and imaging in the same plate without an additional transfer step. We refer to this serum-free one-step protocol as Saponin-Lysis Sexual Stage Assay (SaLSSA). We found SaLSSA to be more sensitive to few compounds like the amino alcohols (see below). Thus, an older, serum-containing assay (two-step sexual stage assay or TSSA) was used in some cases.

#### Assay Evaluation

The quality of the assay was found to be robust at all gametocyte stages: *Z* prime scores calculated with infected, DMSO-treated red blood cells and uninfected red blood cells ranged from 0.71 to 0.80 for the one-step protocols (Figure 1D). We evaluated the fluorescence intensity over time and did not observe significant differences between 30 and 360 min (data not shown).

### Evaluation of Known Antimalarial Drugs Shows Only Few Compounds with Activity against Late-Stage Gametocytes

To further benchmark SaLSSA, we evaluated 50 compounds currently used as antimalarials or antimalarial tool compounds (Delves et al., 2012a) in dose response against individual gametocyte stages. EC_50_ values of different chemical classes showed distinct patterns of activity for the different gametocyte stages as summarized below (Tables 1 and S1). Most compounds yielded higher EC_50_ against stage V gametocytes.

#### Endoperoxides

Endoperoxides were characterized by low nM EC_50_ values for stages I–IV with most failing to generate a dose-response curve for stage Vs, in agreement with standard membrane-feeding data: Although DHA, artesunate, and OZ439 have been reported to have some activity in standard membrane-feeding assays (SMFAs) (Bolscher et al., 2015, Delves et al., 2012a), none completely eliminated oocysts at 100 nM, and only OZ439 eliminated oocysts at 1 μM, a concentration well above the concentrations in blood when being used against blood-stage infections (Bolscher et al., 2015). Interestingly, these results correlate well with previous publications reporting that hemoglobin digestion ends at stages III to IV (Hayward, 2000, Lang-Unnasch and Murphy, 1998), supporting the endoperoxides’ activity against this process (Klonis et al., 2011).

#### 4-Aminoquinolines

The 4-aminoquinolines (chloroquine, piperaquine, pyronaridine, napthoquine, hydroxychloroquine, and AQ13) demonstrated low nM EC_50_ values for stages I and II but showed a drop-off in activity beginning in stage III and were ineffective against stage Vs. It is generally thought that 4-aminoquinolines interfere with the formation of hemozoin, resulting in the death of the parasites, and previous reports have shown that chloroquine is only active against early-stage gametocytes (Sinden, 1982) in line with low activity in SMFAs (Bolscher et al., 2015).

#### 8-Aminoquinolines

The 8-aminoquinolines showed consistent but high (μM) EC_50_ values across all gametocyte stages. Primaquine, the only drug approved for blocking transmission, exhibited a 6.5 μM EC_50_ against stage V gametocytes in vitro. Tafenoquine, a primaquine derivative, had an EC_50_ of 2.4 μM against stage V gametocytes. The substantially higher EC_50_ than the ED_50_ values were expected because 8-aminoquinolines need to be metabolized for activity. The mechanism of action of primaquine as well as the identity of the active metabolites remains unknown.

#### Amino Alcohols

Amino alcohols are not used to prevent transmission, but some amino alcohols (lumefantrine, mefloquine, and halofantrine, but not quinidine and quinine) showed activity across all stages using SaLSSA. We note, however, that this sensitivity could be completely reversed by the addition of human serum to cultures during compound incubation using the TSSA. Mefloquine only shows SMFA activity at high concentrations (10 μM), ∼20× above the asexual growth inhibition values (50 nM). Testing some compounds in the presence of human serum would be expected to give more accuracy and eliminate false positives, but at the expense of efficiency.

#### Other Clinically Relevant Compounds

The antifolates and sulfonamides, which interfere with nucleic acid synthesis and include dihydropteroate synthase (DHPS) and dihydrofolate reductase (DHFR) inhibitors (pyrimethamine, P218, and chlorproguanil), were inactive across all gametocyte stages, except for chlorproguanil, which had μM EC_50_ values against all stages. Antibiotics used to treat malaria, such as doxycycline and azithromycin, which act against the apicoplast, were also inactive. As expected (Fleck et al., 1996), atovaquone was inactive (Table 1). Evidence that this compound has some transmission-blocking activity in animals after repeated exposure (Blagborough et al., 2013) may be because it inhibits ookinete formation (Delves et al., 2012a).

### Antimalarial Compounds with Stage V Activity

Some antimalarial compounds that are not used in humans did show activity against stage V gametocytes in our assay (Table 1). Thiostrepton is a macrocyclic thiopeptide antibiotic that inhibits prokaryotic translation (Harms et al., 2008) and has been reported to dually target the proteasome and apicoplast (Aminake et al., 2011). Thiostrepton had μM EC_50_ values at all stages. The mode of action of methylene blue remains controversial, but it may inhibit glutathione reductase (Buchholz et al., 2008) or hemozoin formation (Adjalley et al., 2011). It showed low nM EC_50_ values for stages I to IV, with some minor loss of activity at stage V, consistent with reports that methylene blue reduced transmission by 99% in SMFAs at 38 nM. Pentamidine, which is clinically used for treatment and prophylaxis of *Pneumocystis carinii* pneumonia (PCP) and sleeping sickness but not malaria, inhibited gametocytes of all stages with an EC_50_ between 0.39 and 2.14 μM. Its mechanism of action is unknown, although it has been reported to inhibit hemozoin formation in *Plasmodium* by interaction with ferriprotoporphyrin IX (Bray et al., 2003, Stead et al., 2001).

### Compounds in Clinical Development

**Figure 2:**
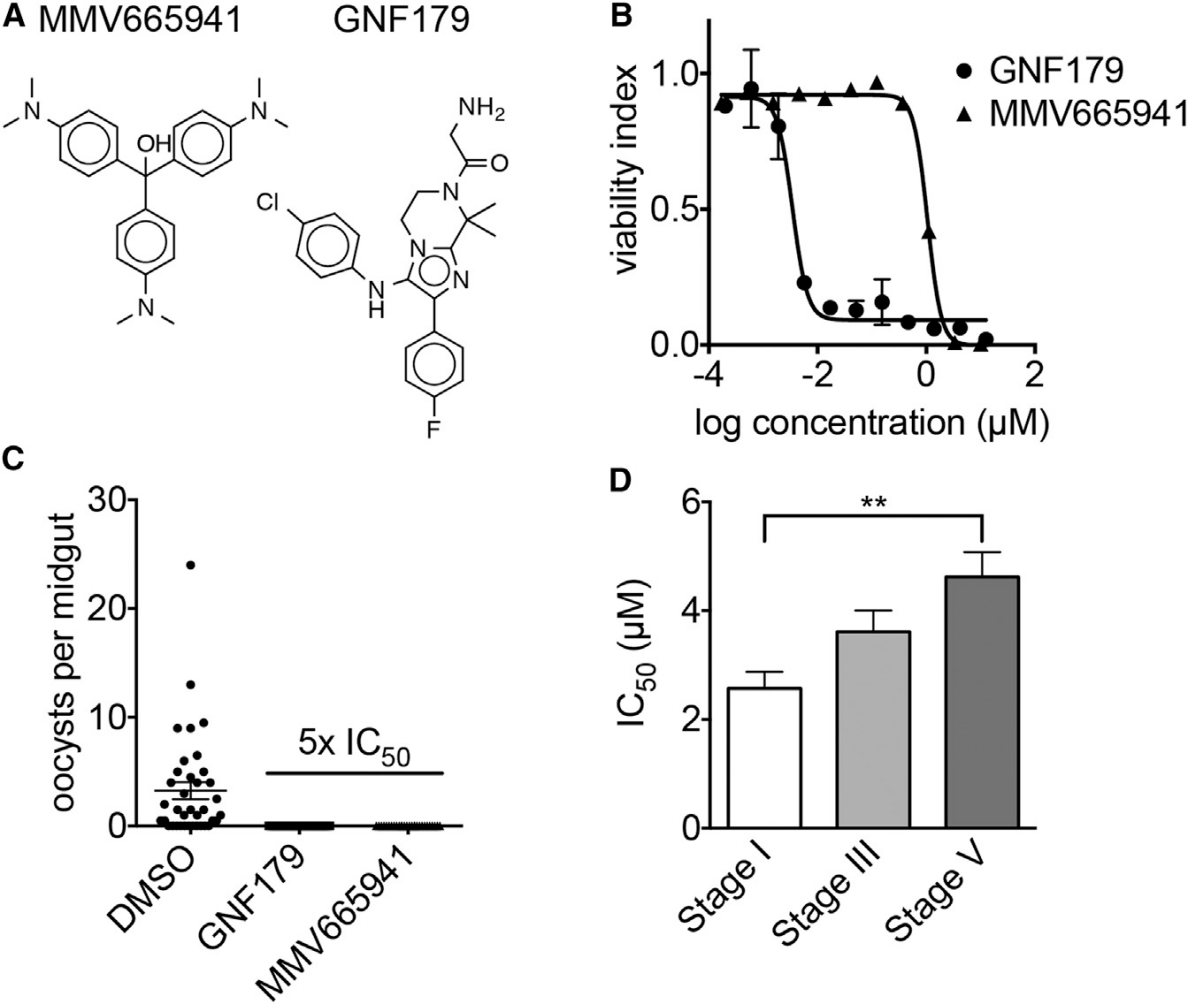
Screening of the MMV Malaria Box in Comparison to GNF179 (A) Structures of MMV665941 and GNF179. (B) In vitro activity of MMV665941 and GNF179 against stage V gametocytes in dose response (1,536-well format, SaLSSA in duplicate). (C) Mean oocyst counts per midgut with gametocytes incubated with DMSO, GNF179 (5× EC_50_), and MMV665941 (5× EC_50_). Experiment was performed in duplicates. (D) Comparison of all 42 EC_50_ values below 12.5μM of the MMV malaria box compounds when screened against gametocytes stages I, III, and V. The average EC_50_ of compounds tested against stage V gametocytes is significantly higher compared to stage I gametocytes (p < 0.05, ANOVA test, Prism 6).

Newer classes of compounds in development, including the spiroindolones (Rottmann et al., 2010), imidazolopiperazines (Meister et al., 2011), imidazopyrazines (McNamara et al., 2013), and quinoline-4-carboxamides (Baragaña et al., 2015), all have reported transmission-blocking activity, and members of these compound classes were tested using SaLSSA (Table 1). KAF246, a spiroindolone closely related to the clinical candidate KAE609 (also known as cipargamin or NITD609) that acts against the plasma membrane ATPase PfATP4 (Rottmann et al., 2010), showed the expected activity (EC_50_ = 1 to 2 nM). GNF179, an imidazopiperazine closely related to the clinical candidate KAF156 (Kuhen et al., 2014), showed the expected low nanomolar activity in the SaLSSA and complete transmission-blocking activity in SMFAs at physiologically relevant concentrations of 15 nM (Figures 2A–2C). The PI(4)K-inhibitor KDU691, which inhibits transmission at 1 μM in SMFAs (McNamara et al., 2013), had submicromolar EC_50_ values across all five gametocyte stages, similar to the values seen against blood stages (∼200 nM). The *Plasmodium falciparum* translation elongation factor 2 (eEF2)-inhibitor, DDD107498, likewise showed potent activity, in line with reported activity in other cellular and standard membrane feeding assays (Baragaña et al., 2015).

### Library 1: MMV Malaria Box Contains Transmission-Blocking Compounds

To determine whether the loss of activity against stage V parasites was typical or reflects the historical focus on compounds derived from quinine and artemisinin, 400 compounds from the MMV malaria box were examined (Spangenberg et al., 2013). These compounds, which were all identified in asexual blood stage screens, were first examined at a single dose of 12.5 μM against each gametocyte stage with TSSA. As expected, the highest number of compounds were active against early-stage gametocytes: 216 compounds inhibited the viability of stage I gametocytes by more than 70%, 78 compounds inhibited stage III gametocytes, and 79 compounds inhibited stage V gametocytes (Table S2).

Dose-response analysis against stage I, III, and V gametocytes for the 50 most active compounds confirmed activity <5 μM for 28 of the 50 compounds with the TSSA. EC_50_ values for stage V were significantly higher than for stage I gametocytes for 42 of the 50 compounds (Figure 2D). A few compounds showed EC_50_ values of ≤1.5 μM against stage V gametocytes in both TSSA and SaLSSA (Table S2), including MMV665941 (stage V EC_50_ 1.04 μM, SaLSSA, Figure 2B) followed by MMV019918 (stage V EC_50_ 1.46 μM, SaLSSA). SMFA studies with MMV665941 using GNF179 as a control at a concentration of five and ten times the EC_50_ calculated from the above described stage V gametocyte assay showed that mosquitoes fed on the compound-exposed gametocytes had no oocysts in their midguts, whereas the DMSO control group did (Figure 2C), most likely because the compound-treated gametocytes did not exflagellate (data not shown).

We further investigated a subset of 18 compounds (Table 2) with reported activity against gametocytes (Bowman et al., 2014, Duffy and Avery, 2013, Ruecker et al., 2014, Sun et al., 2014) and available luciferase-SMFA data using 1,536-well SaLSSA against gametocytes stages I, III, and V. Of this control set, 14 of the 18 were active in at least one stage in SaLSSA with an EC_50_ of less than 10 μM, and 13 of the 14 were active in SMFAs (Figure S3; Table S5). A possible false-negative compound was MMV665882 (Figure S3G), which showed an incomplete curve in SaLSSA but little activity in the SMFA. This compound showed some activity in viability readouts by others (Duffy and Avery, 2013, Sun et al., 2014). The four potential false positives included MMV020492 (Figure S3A), which was previously reported to have low activity against male gametes (Ruecker et al., 2014) but gave no inhibition in SMFAs and was inactive in SaLSSA. Two other false positives were MMV665827 (Figure S3C) and MMV007116 (Figure S3D), which had been shown to reversibly inhibit male gamete formation (Ruecker et al., 2014). The fourth compound, MMV666021 (Figure S3B), which had been reported active in luciferase assays with late-stage gametocytes (Duffy and Avery, 2013), showed some inhibition in the single-point studies but was not reconfirmed in dose response. This compound was weakly active in luciferase-based SMFAs (complete inhibition at 10 μM, partial at 1.6). The reasons for the discrepancies for this compound are unclear, but it is possible that compound source, solubility, or parasite genetic background could play a role, especially as literature-reported blood-stage values (3D7) vary from 90 nM to 2 μM.

### Library 2: GNF Malaria Box

To further investigate the rate at which transmission-blocking compounds would be identified in sets of compounds with known blood-stage activity (EC_50_s of less than 10 μM), we investigated the GNF malaria box (Plouffe et al., 2008). This set of 3,558 compounds was created after screening proliferating asexual parasites at a final compound concentration of 1.25 μM. The set was screened at a single concentration of 1.25 μM against stage V gametocytes with the 384-well SaLSSA. Of these, 145 compounds (4.07%) inhibited stage V gametocytes at greater than 72.3% (Figure S1A). Dose-response analysis showed 108 of the 145 compounds reconfirmed as having activity of less than 1 μM with 22 compounds giving EC_50_ values below 100 nm against stage V gametocytes. Unlike the clinical antimalarials (Table 1), most of which showed a steep drop-off in activity with mature gametocytes, these scaffolds were almost all equipotent against asexual blood stages and stage V gametocytes. Some of the most active scaffolds (Figure S1B) were carbamazide thioureas (Klayman et al., 1979) as well as naphthoquinones, a compound class known to be active against gametocytes (Tanaka et al., 2015). Several of these scaffolds were also active against a *P. yoelii* hepatocyte development and invasion assay (Meister et al., 2011) that predicts causal prophylactic activity (Figure S1C; Table S3).

### Library 3: Broad Diversity-Oriented Synthesis Library

In order to determine the fraction of active compounds that would be found in a larger library that was not preselected for activity against asexual parasites, we tested compounds from the diversity-oriented synthesis (DOS) library (Dandapani and Marcaurelle, 2010). This library was designed to populate chemical space broadly with small molecules having both skeletal and stereochemical diversity (Schreiber, 2000).

Two sets of compounds from the DOS compound library were screened against stage V using 1,536-well SaLSSA at 2.5 μM in duplicate (Figure S2A). The first was an “informer set,” which includes 9,886 compounds selected to represent a sampling of the structural diversity of all of the DOS scaffolds while also capturing preliminary structure-activity relationships (SARs) and stereochemical structure-activity relationships (SSARs). 25 compounds inhibited stage V gametocytes in both replicates by >30% (Table S4, hit rate 0.25%), and 17 were inconclusive (active in one of two replicates). To reconfirm and investigate the SARs as well as SSARs, 41 compounds were retested in dose-response along with 37 stereoisomers and seven analogs of select compounds. 13 of the hits and one inconclusive exhibited EC_50_s < 5 μM upon retest, resulting in a retest rate of 23% (or 54% for hits only).

A second compound set included 89 compounds (representing 17 scaffolds) that had previously been shown to have activity in a blood-stage assay against *P. falciparum* Dd2 (EC_50_ < 2 μM; N.K., unpublished data). These compounds had not been further assessed for mechanism of action or additional stage-specific activity against *Plasmodium* prior to this study. An identical screening pipeline was used for the blood-stage active compounds; in this case, 15 compounds were identified as hits (hit rate 16.9%). These hits and two additional stereoisomers were retested at dose-response, whereupon ten of the compounds exhibited EC_50_s < 5 μM (retest rate 67%).

Taken together, the 35 hits encompassed 12 different scaffolds, with five singletons and seven scaffolds with two or more representatives. Representatives from six of these scaffolds are shown in Figure S2C; one scaffold was eliminated due to a lack of SSARs and SARs in the hits. While the activity needs to be validated with resynthesized compounds, some of these do show interesting patterns of activity, including one compound with greater activity against gametocytes (BRD0608), four compounds with activity across all three parasite stages, and one compound (BRD1260) with activity against just gametocytes and the asexual blood stages (Figure S2B). Additional studies will be of interest to validate these data and investigate the mechanisms of action of these compounds.

### Cheminformatic Compound Clustering

**Figure 3:**
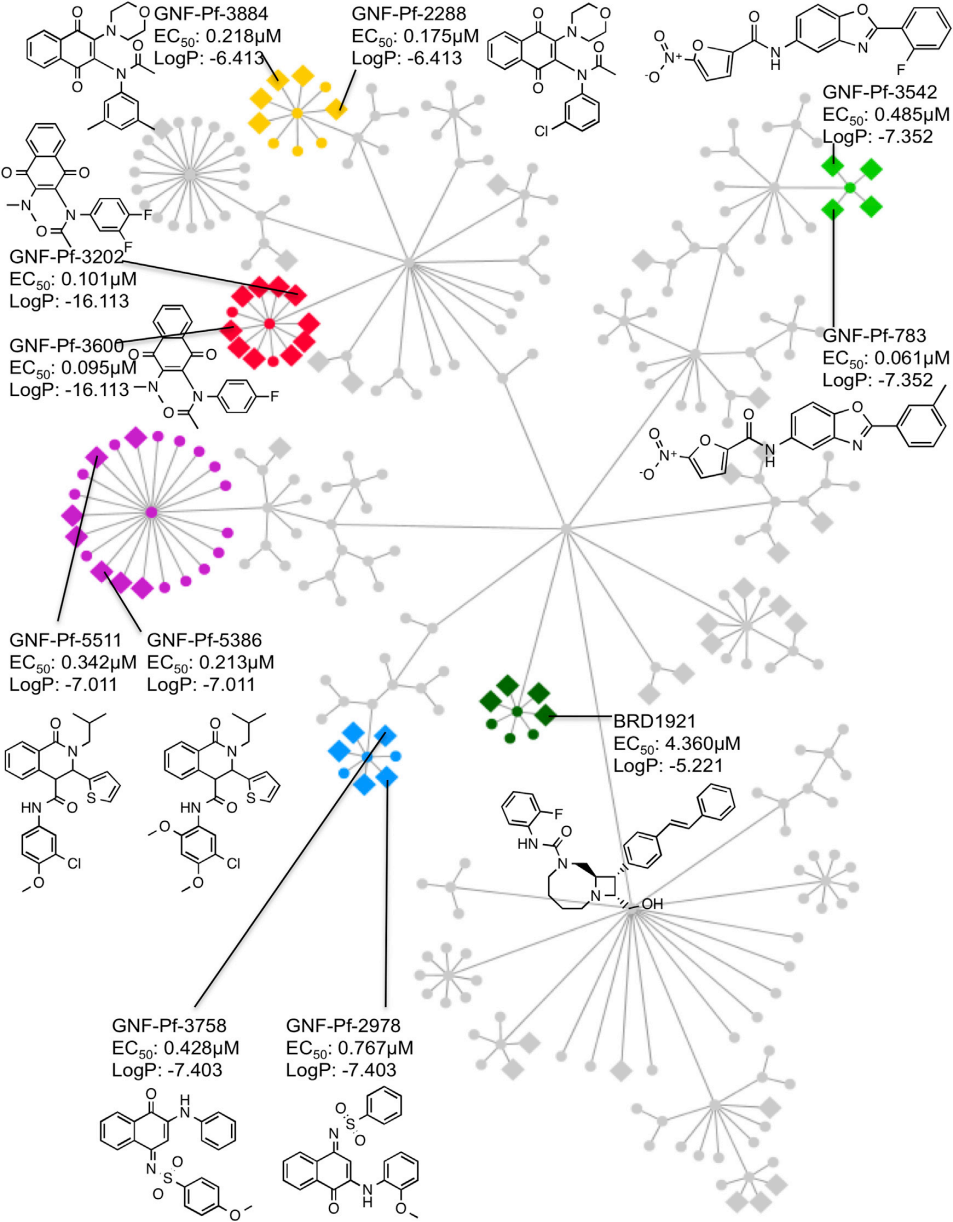
Starting Points for Transmission-Blocking Drugs All compounds in this study were clustered by their substructure similarity. All compounds with the same substructure (Tanimoto average compound similarity ≥ 0.85) were assigned to different scaffold families (indicated by different colors). Non-active compounds are shown as circle nodes and gametocyte-active compounds as diamonds. LogP is log probability of enrichment in the stage V gametocyte active set relative to rate expected by chance for each scaffold family. EC_50_ values are for stage V gametocytes SaLSSA 1,536-well format.

To further validate the screens and to identify compounds that can serve as starting points for the development of the transmission-blocking drugs, all compounds that had been screened were hierarchically clustered based on their scaffold similarity. We then identified clusters of structurally related compounds that showed enrichment in the sexual-stage active set at rates higher than expected by chance (Figure 3). For example, a cluster with the highest enrichment score (enrichment log_10_p = −16.11) consists of 13 compounds related to GNF-Pf-3202 and GNF-Pf-3600 (dioxonapthalen-acetamides), with 10 out of 13 compounds being active in the gametocyte assay. Another enriched cluster contains 21 compounds structurally similar to GNF-Pf-5511 and GNF-Pf-5386 (tetrahydroisoquinoline-4-carboxamide scaffolds that are related to the PfATP4 inhibitor, (+)-dihydroisoquinolones, (+)-SJ733) with seven of the 21 compounds being active (log_10_p = −7.01). This is not unexpected, given that other PfATP4 inhibitors are active against late-stage gametocytes and (+)-SJ733 potently blocks transmission (Jiménez-Díaz et al., 2014). A final scaffold family that is highly overrepresented contains four of the five 2-furancarboxamides (GNF-Pf-1329, GNF-Pf-3542, GNF-Pf-783, GNF-Pf-1696, and GNF-Pf-2740) in the library (log_10_p = −7.35), all of which showed moderate activity against stage V gametocytes (0.61 to 0.484 nM) but weaker activity against asexual blood stage parasites (1.72 to > 10 μM). To our knowledge, this compound class has not previously been associated with blocking transmission. These data suggest that screens of very large libraries will likely yield starting points for the discovery of transmission-blocking drugs.

## Discussion

The present data differ from those that have been reported by a number of other laboratories. Some assays have shown that compounds such as artemether and OZ439 have late-stage gametocytocidal activity of less than 1 μM (Bolscher et al., 2015, Duffy and Avery, 2013). The consensus is that mature gametocytes are resistant to endoperoxides (Delves, 2012, Peatey et al., 2011). Most previous reports combined gametocyte stages for late-stage gametocyte testing (stages III-V or IV-V), which might account for conflicting compound activity (Duffy and Avery, 2013, Lelièvre et al., 2012, Peatey et al., 2011, Sun et al., 2014). Another consideration is that the readout of different gametocytocidal assays might vary with the mode of action of certain drugs, depending on which biological pathway the specific assay is interfering with (Reader et al., 2015).

Overall, our data suggest that low-cost SaLSSA gives few, if any, false positives, compared to available SMFA data. On the other hand, the SaLSSA assay may give a few false-negatives, and will likely miss reversible inhibitors of gamete formation.

In the majority of cases, our data showed that stage V gametocytes had a lower susceptibility to compounds than stage I gametocytes, suggesting decreased metabolic activity during their maturation in preparation for subsequent development in the mosquito midgut. The acquired standard membrane-feeding data, which still represent the gold standard for transmission-blocking activity, suggest that compounds that inhibit stage V gametocytes can block transmission as well. On the other hand, the SMFAs may also find compounds that our cellular assay would miss, including compounds that have a contraceptive effect. Given that fertilization occurs in the mosquito midgut over minutes while late-stage gametocytes can persist in the human body for days, stage V gametocytes are arguably more attractive therapeutic targets.

**Figure 4:**
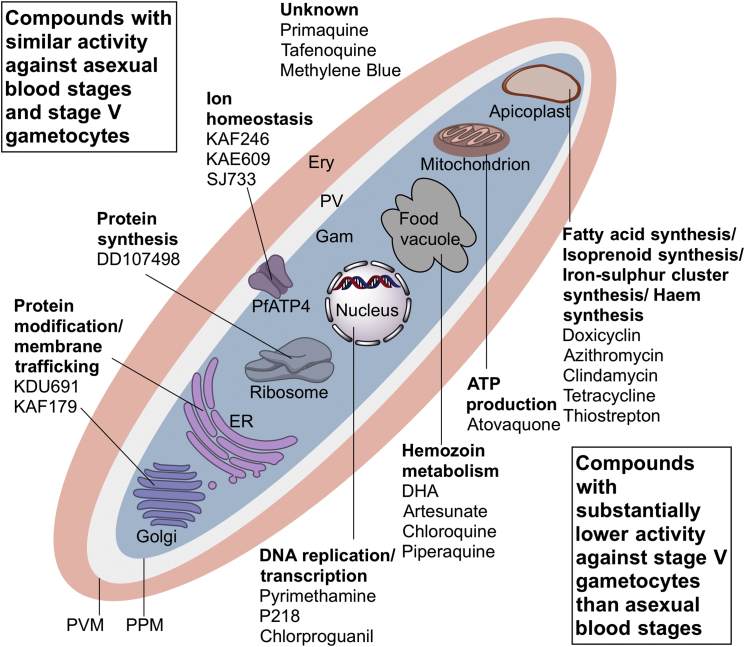
Druggable Organelles and Processes in Gametocytes Infected red blood cell (red) with mature gametocyte (blue) and parasitophorous vacuole (white) as well as schematic parasite organelles known as *P. falciparum* drug targets. The upper (left) section displays compounds with similar activity against stage V gametocytes (SaLSSA) and asexual blood stage parasites as well as their presumed mode of action. The lower (right) section lists compounds with not active against stage V gametocytes compared to asexual blood stage parasites. Ery: erythrocyte; Gam: gametocyte; PPM: parasite plasma membrane; PV: parasitophorous vacuole; PVM: parasitophorous vacuole membrane.

The data from this study indicate which proteins and pathways might be targeted by transmission-blocking drugs (Figure 4). Compounds that interfere with hemoglobin digestion would be poor candidates for transmission-blocking drugs, although they could yield a reduction in gametocyte numbers as early-stage gametocytes might be killed. In addition, the process of DNA replication should probably not be targeted, nor apicoplast function (azithromycin and doxycycline). Targets for compounds that act against mature gametocytes include proteins that play a role in protein translation (the targets of puromycin, DDD107498, cycloheximide, thiostreptin) and processing, including protein secretion (GNF179), as well as protein degradation (e.g., epoxomycin; Czesny et al., 2009). Interestingly, functional genomic studies had previously shown that gametocytes acquire and store RNA transcripts that rapidly convert to proteins during gamete formation (Mair et al., 2006), creating a particular vulnerability. Targets involved in maintaining ion homeostasis, such as PfATP4 as well as lipid kinases (e.g., PI4K), are transmission-blocking targets as well as asexual stage targets.

It should be noted that all these targets are also essential for asexual parasites. Targeting exclusively gametocytes could be achieved through inhibiting translational repression, autophagy, sperm function, as well as meiosis. It is expected that compounds inhibiting these processes would be found at lower rates in large libraries, emphasizing the need for ultra-high-throughput screens. One example is BRD0608, whose EC_50_ against asexual blood stages was 15× higher than against stage V gametocytes and whose selectivity for sexual stages might be improved through medicinal chemistry.

The advantage of compounds like BRD0608 is a reduced potential for emergence of drug resistance. There are billions of asexually replicating parasites in an infected human, each of which has the capacity to develop a drug resistance mutation and pass it on to their progeny. This has been a major reason why malaria control is so difficult.

An open ethical question is whether drugs, which do not relieve malaria symptoms but benefit the community as a whole, should be licensed. Vaccines may be given that provide little benefit to an individual—for example, the rubella vaccine in boys, mainly recommended to cohort-protect pregnant women in order to prevent congenital rubella syndrome of newborns. Vaccines are also not without risk, and one could argue that the benefit to humanity that would be achieved with malaria eradication would outweigh the risk.

## Experimental Procedures

### Gametocyte Culture

Asexual *P. falciparum* parasites (NF54) were grown at 5% hematocrit in O+ human erythrocytes in serum-containing complete media (RPMI 1640, gentamicin 0.05 mg/ml, hypoxanthine 0.014 mg/ml, HEPES 38.4 mM, sodium bicarbonate 0.2% [w/v], D-glucose 0.2% [w/v], sodium hydroxide 3.4 mM, 4.3% [w/v] heat-inactivated human serum [O+] and 0.2% [w/v] AlbuMAX II) at 37°C under low-oxygen conditions (3% O_2_, 5% CO_2_, and 92% N_2_) and a parasitemia between 0.5% and 3%. Ring-stage parasites were triple synchronized (d-8, d-6, d-4) with 5% (w/v) D-Sorbitol, and cultures were expanded from T25 to T225 culture flasks. The hematocrit was adjusted to 5% until day −4. On day −2, only 50% fresh media was substituted during a high parasitemia of 7%–10%. Media was exchanged daily from day −1 onward. For stages I–IV, magnetically activated cell sorting (MACS) was performed on day 0 and cultures were sorbitol synchronized on day 1. All gametocytes were treated with 50 mM NAG on days 0–9. See Supplemental Experimental Procedures for more details.

### Compound Assays

Gametocyte stages I–V were diluted to 0.50% gametocytemia and 1.25% hematocrit into complete media for the two step protocol (TSSA) or 0.5%–0.75% gametocytemia and 1.25% hematocrit into serum-free SALSSA screening media (RPMI 1640, gentamicin 0.05 mg/ml, hypoxanthine 0.014 mg/ml, HEPES 38.4 mM, sodium bicarbonate 0.2% [w/v], D-glucose 0.2% [w/v], sodium hydroxide 3.4mM and 0.4% [w/v] AlbuMAX II). Cultures were dispensed (40 μl versus 10 μl) into 384 or 1,536-well plates containing 50 nl or 2.5 nl of compound (final concentration of 1.25 to 12.5 μM) using a MultiFlo dispenser. Plates were incubated at 37°C for 72 hr under low-oxygen conditions. For SaLSSA 3 μl (1,536 well) or 10 μl (384 well) of 2.5 μM MitoTracker Red CMXRos and 0.13% saponin solution (w/v) in screening media was added to each well, and plates were incubated for 60–120 min at 37°C. For 384-well TSSA, 5 μl MitoTracker Red CMXRos (5 μM) in screening media was added to each well. After 20 min at 37°C, 5 μl was transferred from the assay plate to a new 384-well imaging plate, that already contained 40 μl MitoTracker Red CMXRos (500nm) in serum-free screening media. For both TSSA and SaLSSA, plates were imaged after 30 min incubation.

### High-Content Imaging and Analysis

Imaging of 384- or 1,536-well plates was performed using a high content imaging system (Operetta, Perkin Elmer) and Harmony software for image analysis. Viability indices were calculated by dividing the particle count of each compound-treated well by the average particle count of the DMSO wells per plate and range from 0 (active compound) to >1 (inactive). *Z* values were calculated using DMSO-treated gametocytes as positive and uninfected red blood cells as negative wells.

### SMFA—Traditional

*P. falciparum* NF54 parasites were grown at 0.5% parasitemia and 5% hematocrit and continuously cultured with daily media changes until they reached stage V (Gregory et al., 2012). After incubation for 24 hr with compound in DMSO, the SMFA was performed (Gregory et al., 2012). Briefly, 4- to 6-day-old female *A. stephensi* STE 2 mosquitoes were fed with the treated gametocytes for 15 min using a membrane feeding apparatus. Midguts were dissected after 8 days, and the number of oocysts counted.

### SMFA—Luciferase

*P. falciparum* NF54-L1 (hsp70-luc reporter) stage V gametocytes were pre-incubated for 24 hr with compound in six dilutions in duplicate. DMSO was used as negative and DHA as positive control. The compound was washed out, and the gametocytes were fed to *Anopheles stephensi* mosquitoes. At day 8 post-infection, luminescence signals were determined for 24 individual mosquitoes per cage. EC_50_s were determined by applying a four parameter logistic regression model. The SMFAs, which were organized by a consortium of laboratories including this one, have been deposited at ChEMBL-NTD (https://www.ebi.ac.uk/chemblntd) and were performed by TropIQ in Nijmegen, The Netherlands.

### *P. berghei* Liver Stage Invasion Assay

Liver stage assays were performed as previously described (Baragaña et al., 2015). Briefly, 10^3^ *P. berghei* luciferase-expressing sporozoites (New York University Insectary) were used to infect HepG2-A16-CD81^EGFP^ cells (pretreated with compound at 1.25 μM) in a 1,536-well plate. After incubation for 48 hr, 2 μl BrightGlo (Promega) was added, and the EEF growth was quantified by bioluminescence on an Envision Multilabel Reader (PerkinElmer).

### Compound Clustering

13,844 tested compounds were clustered using the Scaffold Tree algorithm (Schuffenhauer et al., 2007). Each scaffold node was then assigned an enrichment score reflecting the degree of overrepresentation of active compounds (stage V gametocyte inhibitors). We calculated the accumulative hypergeometric p value as probability of observing at least as many hits as we observed within each scaffold. The tree was then pruned, so that only scaffolds with p values < 0.001 were retained. The final resultant tree in Figure 3 was rendered with Cytoscape (version 3.2.0). To focus on those nodes where the scaffold of a node could reasonably resemble the full structures of all associated compound members, the average Tanimoto similarity score between each scaffold node and its associated compounds were calculated based on ChemAxon topological fingerprints (ChemAxon, Kft.), and those tree nodes and leaves with at least 0.85 Tanimoto scores and with at least three hits are highlighted in colors in Figure 3.

## Author Contributions

D.M.P. and M.W. designed experiments, performed screens and dose response assays, analyzed data, and wrote the manuscript; A.Y.D. prepared parasite materials; S.M. performed *P. berghei* liver stage assay; F.L., K.P., and A.L. performed standard membrane feeding assays; S.L.O. designed experiments; E.L.F. analyzed data; O.T. and Y.Z. performed compound clustering; E.C. performed data analysis; N.K. performed Dd2 blood stage assays; C.A.S. assisted with experimental design analyzed data; S.L.S. and D.L. performed data analysis; and E.A.W. designed experiments, analyzed data, and wrote manuscript. All authors edited the manuscript and contributed to writing.

## Figures and Tables

**Table 1:** Well-Characterized Antimalarials Active against Asexual Blood Stages Tested in Dose Response against the Five Gametocyte Stages

Compound	Chemical Class	EC_50_ (μM) ± STD					Oocyst Reduction
Stage I	Stage II	Stage III	Stage IV	Stage V
Artemisinin	Endoperoxide	0.024 ± 0.001	0.020 ± 0.010	0.012 ± 0.002	0.037 ± 0.003	>12.500 ± 0.000	
Artemisone	Endoperoxide	0.003 ± 0.000	0.002 ± 0.000	0.003 ± 0.000	0.004 ± 0.001	>12.500 ± 0.000	
Artemether	Endoperoxide	0.016 ± 0.006	0.005 ± 0.001	0.006 ± 0.002	0.019 ± 0.004	>12.500 ± 0.000	10 μM: 75%–99%^3^
Artenimol (Dihydroartemisinin)	Endoperoxide	0.006 ± 0.001	0.003 ± 0.001	0.007 ± 0.000	0.021 ± 0.008	>12.500 ± 0.000	1 μM: ∼90%^1^
Artesunate	Endoperoxide	0.008 ± 0.002	0.004 ± 0.000	0.013 ± 0.003	0.049 ± 0.013	>10.601 ± 2.686	1 μM: ∼55%^1^
OZ439 mesylate	Endoperoxide	0.011 ± 0.001	0.005 ± 0.000	0.003 ± 0.000	0.002 ± 0.000	>12.500 ± 0.000	1 μM: 100%^1^
OZ277(RBX-11160)	Endoperoxide	0.008 ± 0.001	0.004 ± 0.000	0.002 ± 0.000	0.008 ± 0.003	>12.500 ± 0.000	10 μM: 75%–99%^3^
Amodiaquine	4-aminoquinoline	0.012 ± 0.001	0.006 ± 0.001	0.096 ± 0.013	2.456 ± 0.757	1.783 ± 0.119	10 μM: 50%–74%^3^
AQ-13	4-aminoquinoline	0.043 ± 0.002	0.033 ± 0.000	0.484 ± 0.159	6.471 ± 0.888	6.051 ± 1.091	10 μM: 25%–49%^3^
Chloroquine	4-aminoquinoline	0.096 ± 0.007	0.098 ± 0.005	>6.250 ± 0.000	>6.250 ± 0.000	>6.25 ± 0.000	10 μM: 25%–49%^3^
Hydroxychloroquine	4-aminoquinoline	0.107 ± 0.011	0.131 ± 0.005	>4.489 ± 2.490	>6.250 ± 0.000	>6.25 ± 0.000	10 μM: 25%–49%^3^
Naphthoquine	4-aminoquinoline	0.025 ± 0.003	0.014 ± 0.000	0.296 ± 0.078	>4.167 ± 0.000	>4.167 ± 0.000	
Piperaquine phosphate	4-aminoquinoline	0.018 ± 0.002	0.014 ± 0.003	0.031 ± 0.007	>4.167 ± 0.000	>4.167 ± 0.000	1 μM: ∼25%^1^
Pyronaridine phosphate	4-aminoquinoline	0.013 ± 0.001	0.010 ± 0.002	0.125 ± 0.079	2.579 ± 0.811	2.075 ± 0.031	1 μM: ∼80%^1^
Primaquine	8-aminoquinoline	2.467 ± 0.168	2.254 ± 0.236	5.400 ± 1.471	5.151 ± 0.253	6.500 ± 0.955	
NPC-1161B	8-aminoquinoline	2.002 ± 0.188	2.023 ± 0.288	3.510 ± 0.013	3.599 ± 0.182	2.865 ± 0.031	10 μM: 100%^3^
Pamaquine (diethlyprimaquine)	8-aminoquinoline	1.682 ± 0.318	1.404 ± 0.000	2.411 ± 0.672	2.824 ± 0.250	2.529 ± 1.033	
Tafenoquine	8-aminoquinoline	4.560 ± 0.942	3.682 ± 0.871	3.738 ± 0.210	3.484 ± 0.300	2.449 ± 0.262	
Mefloquine (+ RS)	Amino alcohol∗	0.038 ± 0.001	0.039 ± 0.005	0.078 ± 0.013	0.579 ± 0.600	0.158 ± 0.007	10 μM: 100%^3^
Halofantrine	Amino alcohol∗	0.002 ± 0.001	0.001 ± 0.000	0.023 ± 0.039	1.50946 ± 2.072	0.007 ± 0.002	10 μM: 75%–99%^3^
Lumefantrine	Amino alcohol∗	0.013 ± 0.002	0.013 ± 0.002	0.015 ± 0.006	0.599 ± 0.188	0.052 ± 0.016	1 μM: ∼60%^1^
Mefloquine (Racemic)	Amino alcohol∗	0.044 ± 0.001	0.052 ± 0.006	0.143 ± 0.059	0.818 ± 0.005	0.132 ± 0.003	
Quinidine	Amino alcohol∗	0.138 ± 0.034	0.208 ± 0.007	>9.167 ± 4.714	>12.500 ± 0.000	>11.832 ± 0.945	
Quinine sulfate dihydrate	Amino alcohol∗	0.440 ± 0.039	0.496 ± 0.115	>8.775 ± 5.268	>12.500 ± 0.000	>8.865 ± 5.141	
Methylene Blue trihydrate	Aromatic	0.020 ± 0.008	0.015 ± 0.005	0.013 ± 0.001	0.012 ± 0.002	0.258 ± 0.029	38 nM: 99%^2^
Thiostrepton	Antibiotic	3.405 ± 0.157	3.373 ± 0.262	2.837 ± 0.232	1.820 ± 0.419	3.261 ± 0.461	
Azithromycin	Antibiotic	>12.500 ± 0.000	>12.500 ± 0.000	>12.236 ± 0.374	>12.500 ± 0.000	>12.500 ± 0.000	10 μM: 0%^3^
Doxycyclin	Antibiotic	>12.500 ± 0.000	>12.500 ± 0.000	>12.500 ± 0.000	>12.500 ± 0.000	>12.500 ± 0.000	10 μM: 0%^3^
Trimethoprim	Antibiotic	>12.500 ± 0.000	>12.500 ± 0.000	>12.500 ± 0.000	>12.500 ± 0.000	>12.500 ± 0.000	
Cis-Mirincamycin (HCl)	Antibiotic	>12.500 ± 0.000	>12.500 ± 0.000	>12.500 ± 0.000	>12.500 ± 0.000	>12.500 ± 0.000	
Trans-Mirincamycin (HCl)	Antibiotic	>12.500 ± 0.000	>12.500 ± 0.000	>12.500 ± 0.000	>12.500 ± 0.000	>12.500 ± 0.000	
Fosmidomycin mono sodium	Antibiotic	>12.500 ± 0.000	>12.500 ± 0.000	>12.500 ± 0.000	>12.500 ± 0.000	>12.500 ± 0.000	
Clindamycin	Antibiotic	>12.500 ± 0.000	>12.500 ± 0.000	>12.500 ± 0.000	>12.500 ± 0.000	>12.500 ± 0.000	
Tetracycline	Antibiotic	>12.500 ± 0.000	>12.500 ± 0.000	>12.500 ± 0.000	>12.500 ± 0.000	>12.500 ± 0.000	
Chlorproguanil hydrochloride	Antifolate	9.350 ± 1.506	7.794 ± 0.287	7.869 ± 0.177	5.209 ± 1.107	4.798 ± 0.758	
Dapsone	Antifolate	>12.500 ± 0.000	>12.500 ± 0.000	>12.500 ± 0.000	>12.500 ± 0.000	>12.500 ± 0.000	
Pyrimethamine	Antifolate	>12.500 ± 0.000	>12.500 ± 0.000	>12.500 ± 0.000	>12.500 ± 0.000	>12.500 ± 0.000	
Cycloguanil	Antifolate	>12.500 ± 0.000	>12.500 ± 0.000	>12.500 ± 0.000	>12.500 ± 0.000	>12.500 ± 0.000	
Proguanil hydrochloride	Antifolate	>12.500 ± 0.000	>12.500 ± 0.000	>12.500 ± 0.000	>12.500 ± 0.000	>12.500 ± 0.000	
P218.HCl	Antifolate	>12.500 ± 0.000	>12.500 ± 0.000	>12.500 ± 0.000	>12.500 ± 0.000	>12.500 ± 0.000	
Sulfadiazine	Sulfonamide	>12.500 ± 0.000	>12.500 ± 0.000	>12.500 ± 0.000	>12.500 ± 0.000	>12.500 ± 0.000	
Sulfamethoxazole	Sulfonamide	>12.500 ± 0.000	>12.500 ± 0.000	>12.500 ± 0.000	>12.500 ± 0.000	>12.500 ± 0.000	
Sulfadoxine	Sulfonamide	>12.500 ± 0.000	>12.500 ± 0.000	>12.500 ± 0.000	>12.500 ± 0.000	>12.500 ± 0.000	
Cycloheximide	Other	1.917 ± 0.060	0.640 ± 0.028	0.913 ± 0.027	0.477 ± 0.402	2.692 ± 0.080	
Pentamidine	Other	0.397 ± 0.040	0.591 ± 0.027	0.697 ± 0.016	0.813 ± 0.177	2.143 ± 0.189	
Dehydroepiandrosterone sulfate	Other	>12.500 ± 0.000	>12.500 ± 0.000	>12.500 ± 0.000	>12.500 ± 0.000	>12.500 ± 0.000	
Flavin mononucleotid (Riboflavin)	Other	>12.500 ± 0.000	>12.500 ± 0.000	>12.500 ± 0.000	>12.500 ± 0.000	>12.500 ± 0.000	
N-acetyl-D-penicillamine	Other	>12.500 ± 0.000	>12.500 ± 0.000	>12.500 ± 0.000	>12.500 ± 0.000	>12.500 ± 0.000	
Deferoxamine mesylate salt	Other	>12.500 ± 0.000	>12.500 ± 0.000	>12.500 ± 0.000	>12.500 ± 0.000	>12.500 ± 0.000	
Puromycin	Control	0.123 ± 0.069	0.122 ± 0.045	0.103 ± 0.039	0.110 ± 0.038	0.122 ± 0.048	
Atovaquone	Naphthalene	>12.500 ± 0.000	>12.500 ± 0.000	>12.500 ± 0.000	2.373 ± 1.995	>12.500 ± 0.000	
GNF179	Imidazolopiperazine	0.341 ± 0.090	0.064 ± 0.017	0.020 ± 0.000	0.009 ± 0.005	0.003 ± 0.001	15 nM: 100%^5^
KAI407	Imidazopyrazine	0.593 ± 0.079	0.636 ± 0.057	0.415 ± 0.073	0.329 ± 0.043	0.156 ± 0.026	
KDU691	Imidazopyrazine	0.532 ± 0.024	0.565 ± 0.009	0.354 ± 0.071	0.237 ± 0.057	0.150 ± 0.001	1 μM: 100%^4^
KAF246	Spiroindolone	0.001 ± 0.000	0.002 ± 0.000	0.002 ± 0.000	0.002 ± 0.001	0.002 ± 0.001	
DDD107498	Quinoline-4-carboxamide	0.003 ± 0.000	0.005 ± 0.002	0.005 ± 0.001	0.002 ± 0.000	0.009 ± 0.002	EC_50_: 1.8 nm^6^
DMSO	Control	>12.500 ± 0.000	>12.500 ± 0.000	>12.500 ± 0.000	>12.500 ± 0.000	>12.500 ± 0.000	

EC_50_ is displayed in μM ± SD (SaLSSA, 384 well in duplicate).

∗ Amino alcohols (lumefantrine) showed little transmission-blocking activity in the presence of human serum (see Table S1).

1 Oocyst reductions derived from Bolscher et al. (2015).

2 Oocyst reductions derived from Adjalley et al. (2011).

3 Oocyst reductions derived from Delves et al. (2012a).

4 Oocyst reductions derived from McNamara et al. (2013).

5 Oocyst reductions derived from this manuscript.

6 Oocyst reductions derived from Baragaña et al. (2015).

**Table 2:** Activity of 18 MMV Control Compounds against Sexual and Asexual Stages

Compound	Viability INDEX					EC_50_ (μM)			
Stage I	Stage II	Stage III	Stage IV	Stage V	Stage Asex	Stage I	Stage III	Stage V
MMV000442	0.120	0.192	0.385	0.633	0.442	0.362^a^	0.553 ± 0.041	>10.000 ±0.000	>10.000 ±0.000
MMV665971	0.056	0.097	0.137	0.171	0.416	0.489^a^	0.746 ± 0.116	>10.000 ±0.000	>10.000 ±0.000
MMV011438	0.005	0.005	0.015	0.000	0.000	0.327^a^, 0.332^b^	1.120 ± 0.116	2.428 ±0.620	4.225 ±0.609
MMV000248	0.006	0.057	0.061	0.085	0.121	0.719^a^	1.058 ± 0.026	3.074 ±0.533	3.700 ±0.173
MMV666125	0.006	0.002	0.071	0.196	0.234	0.381^a^, 2.844^b^	0.094 ± 0.068	1.342 ±0.259	6.154 ±0.569
MMV019918	0.017	0.014	0.010	0.015	0.033	0.800^a^	1.264 ± 0.145	0.576 ±0.069	1.463 ±0.296
MMV019266	0.028	0.014	0.035	0.082	0.066	0.615^a^	0.935 ± 0.214	1.372 ±0.210	1.743 ±0.206
MMV396797	0.034	0.007	0.033	0.094	0.077	0.477^a^	5.435 ± 0.470	2.094 ±0.424	3.477 ±0.175
MMV667491	0.037	0.048	0.034	0.032	0.000	1.230^a^	0.980 ± 0.069	0.667 ±0.101	0.596 ±0.085
MMV019881	0.063	0.065	0.039	0.047	0.013	0.646^a^	3.048 ± 3.278	8.408 ±1.577	0.721 ±0.141
MMV000448	0.066	0.016	0.083	0.201	0.231	0.235^a^, 0.033^b^	1.195 ± 0.113	5.356 ±0.612	4.652 ±0.394
MMV665882	0.116	0.092	0.122	0.168	0.130	0.466^a^	0.180 ± 0.036	1.477 ±0.136	0.984 ±0.022
MMV665941	0.157	0.020	0.095	0.061	0.110	0.255^a^	0.388 ± 0.035	2.271 ±0.751	1.044 ±0.040
MMV665980	0.240	0.184	0.087	0.108	0.247	0.211^b^	>10.000 ± 0.000	9.350 ±3.237	6.612 ±1.100
MMV007116	0.377	0.505	0.734	0.733	0.846	0.351^a^, 0.716^b^	>10.000 ± 0.000	>10.000 ±0.000	>10.000 ±0.000
MMV665827	0.592	0.900	0.810	0.769	0.791	0.119^a^, 0.166^b^	>10.000 ± 0.000	>10.000 ±0.000	>10.000 ±0.000
MMV666021	0.610	0.871	0.740	0.897	1.055	0.094^a^, 1.998^b^	>10.000 ± 0.000	>10.000 ±0.000	>10.000 ±0.000
MMV020492	0.768	0.756	0.970	0.912	0.989	0.026^a^	>10.000 ± 0.000	>10.000 ±0.000	>10.000 ±0.000

The viability index shows the ratio for each gametocyte stage (I–V) compared to DMSO-treated control wells screened at 12.5 μM (384-well TSSA). EC_50_ values for stage I, III, and V, gametocytes are displayed as mean ± SD (1,536-well SaLSSA, in duplicate). Asexual stage data for 3D7 parasites as provided by MMV and the ChEMBL-NTD repository (https://www.ebi.ac.uk/chemblntd).

a 72 hr DAPI assay (Duffy and Avery, 2012).

b 72 hr SYBR green assay (Meister et al., 2011).

## Supplemental Information

Document S1. Figures S1–S3, Table S5, and Supplemental Experimental Procedures

Table S1. MMV Control Compounds and Known Antimalarials

Table S2. MMV Malaria Box Compounds

Table S3. GNF Malaria Box Compounds

Table S4. Broad DOS Compounds

Document S2. Article plus Supplemental Information
