# Supplementary material for: High-Throughput Assay and Discovery of Small Molecules that Interrupt Malaria Transmission
Source: Cell Host Microbe. 2016 Jan 13;19(1):114–26. doi: 10.1016/j.chom.2015.12.001 (PMC4723716; doi:10.1016/j.chom.2015.12.001)
Supplement: Document S1. Figures S1–S3, Table S5, and Supplemental Experimental Procedures [file mmc1.pdf]

Cell Host & Microbe, Volume *19*

Supplemental Information

High-Throughput Assay and Discovery of Small Molecules that Interrupt Malaria Transmission

David M. Plouffe, Melanie Wree, Alan Y. Du, Stephan Meister, Fengwu Li, Kailash Patra, Aristea Lubar, Shinji L. Okitsu, Erika L. Flannery, Nobutaka Kato, Olga Tanaseichuk, Eamon Comer, Bin Zhou, Kelli Kuhen, Yingyao Zhou, Didier Leroy, Stuart L. Schreiber, Christina A. Scherer, Joseph Vinetz, and Elizabeth A. Winzeler

Figure S1

A

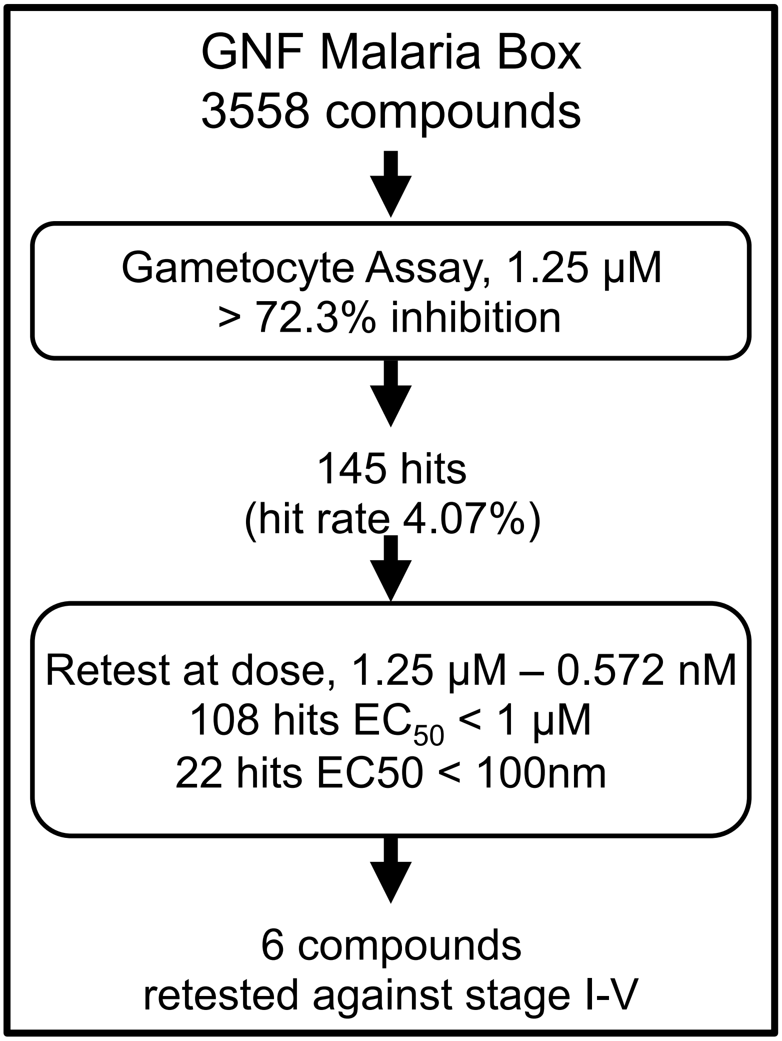

B

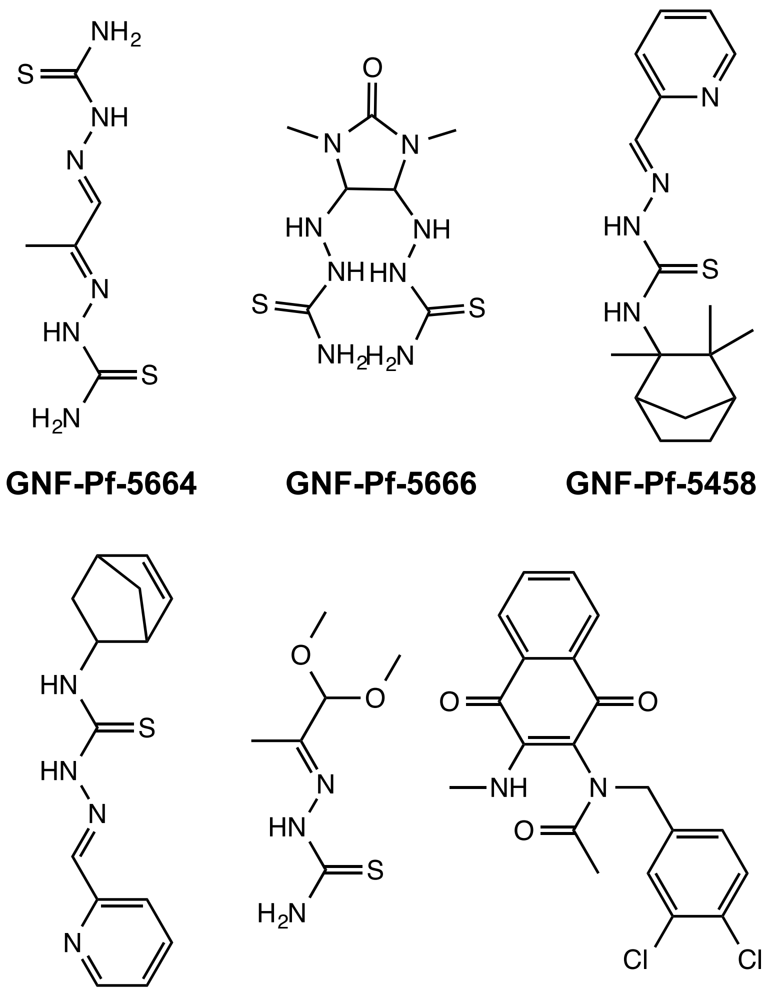

C

	**GNF-Pf-5664**	**GNF-Pf-5666**	**GNF-Pf-5458**	**GNF-Pf-5349**	**GNF-Pf-5675**	**GNF-Pf-3788**
Gametocyte (Stage V) EC_{50}(µM)	0.004 ± 0.0004	0.014 ± 0.002	0.002 ± 0.0003	0.004 ± 0.0004	0.007 ± 0.00004	0.005 ± 0.0013
Blood stage (3D7) EC50 (µM)	0.009	0.009	0.035	0.039	0.009	0.001
Liver stage (*P. yoelii*) EC50 (µM)	n/a	0.015	0.01738	n/a	0.00493	n/a
Huh7 CC50 (µM)	>100	>100	54.71	44.32	>100	0.126

Figure S1, related to Figure 3 and Table S3. Transmission-blocking compounds from the GNF malaria box
A) Screening pipeline for the 3558 GNF malaria box compounds. The initial hit rate was 4.07% when stage V gametocytes were assayed at 1.25µM (SaLSSA, 384-well format). B) Chemical structures for six amongst the most potent gametocytocidal compounds are shown. C) Representative compounds with EC_{50} against stage V gametocytes (SaLSSA, 384-well in duplicate) and their activity against asexual blood stages (*P. falciparum* 3D7) (Meister et al., 2011, Plouffe et al., 2008), liver stage (*P. yoelii*–HepG2) (Meister et al., 2011), and mammalian cell cytotoxicity (Meister et al., 2011).

Figure S2

A

1. Informer Set
9,886 compounds

↓

Gametocyte Assay, 2.5 μM
> 30% inhibition

↓

25 hits
17 inconclusive
(hit rate 0.25%)

↓

Retest at dose, 10 μM – 0.5 nM
41 hits (hits + inconclusive)
37 stereoisomers + 7 analogs
EC_{50} < 5 μM

↓

14 compounds
retest rate 23%

2. Blood Stage Actives
89 compounds

↓

Gametocyte Assay, 2.5 μM
> 30% inhibition

↓

15 hits
0 inconclusive
(hit rate 16.9%)

↓

Retest at dose, 10 μM – 0.5 nM
15 hits
2 stereoisomers
EC_{50} < 5 μM

↓

10 compounds
retest rate 67%

B

	BRD0608	BRD3456	BRD1160	BRD1260	BRD4696	BRD9243
Gametocyte (Stage V) EC_{50}(μM)	0.57, 0.41	0.51, 0.17	1.83, 1.65	1.73, 1.29	3.93	2.7
Blood stage (Dd2) EC50 (μM)	7.3	0.15	0.24	4.2	0.27	0.47
Liver stage (*P. berghei*) EC_{50} (μM)	> 10	0.07	5.06	> 10	0.73	3.62
HepG2 CC50 (μM)	> 10	> 10	>10	> 10	>10	>10

C

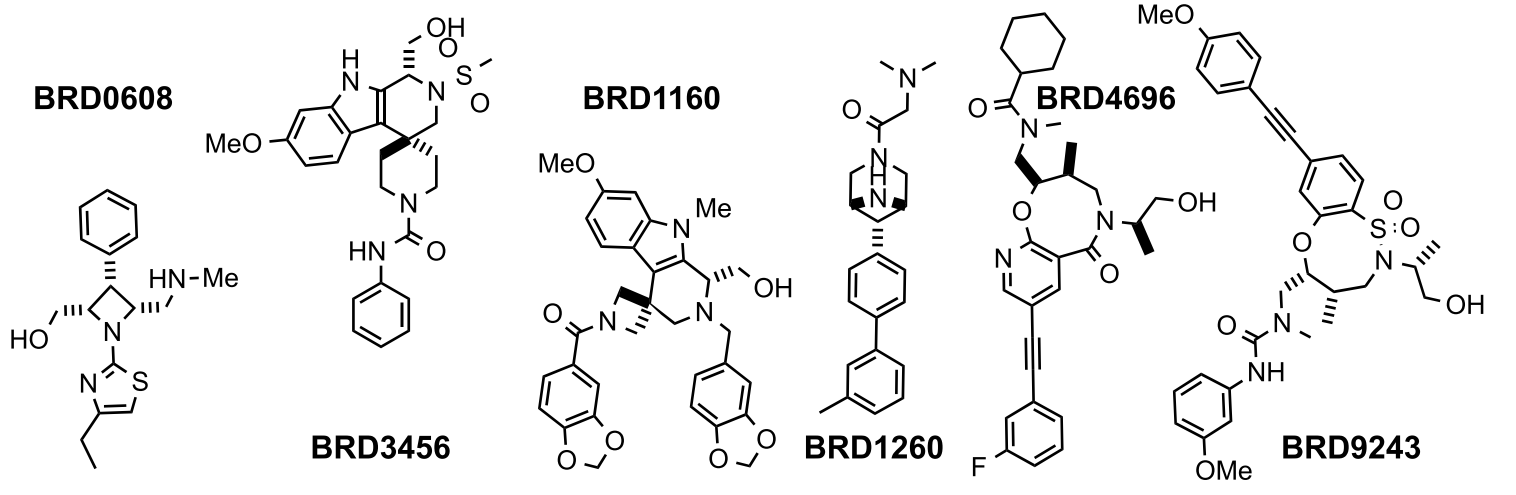

Figure S2, related to Figure 3 and Table S4. Gametocytocidal compounds from the Broad DOS Library

A) Screening pipeline for naïve informer set compounds and pre-selected blood-stage active compounds. The hit rate for the naïve compound set was 0.25%, while the hit rate for the pre-selected compounds was 16.9% when stage V gametocytes were assayed at 2.5 μM with SaLSSA in 1536-well format. B) Representative compounds and their activity profiles with activity against stage V gametocytes in the 1536-well SaLSSA assay. Compounds were also retested in blood stage (*P. falciparum* Dd2), liver stage (*P. berghei*–HepG2), and mammalian cell cytotoxicity assays in dose response; some compounds were tested in dose response in the gametocyte assay twice; both values are shown. C) Chemical structures for the six representative compounds are shown.

Figure S3

Compound		SaLSSA stage V EC_{50}	Reported activity (EC_{50})
A) MMV020492	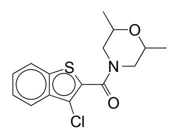	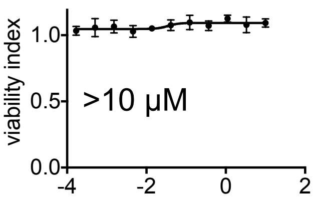 >10 µM	Blood stage: 0.089 µM SMFA: not active
B) MMV666021	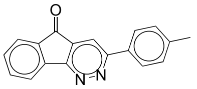	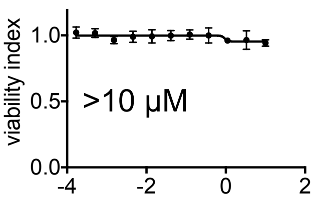 >10 µM	Blood stage: 1.99 µM SMFA: 1.26 µM
C) MMV665827	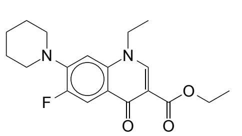	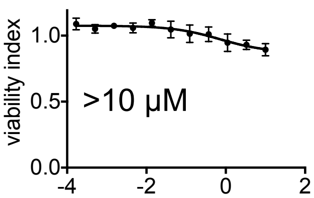 >10 µM	Blood stage: 0.166 µM SMFA: 0.10 µM
D) MMV007116	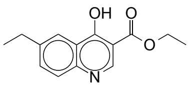	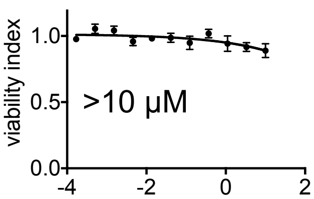 >10 µM	Blood stage: 0.716 µM SMFA: 0.11 µM
E) MMV019918	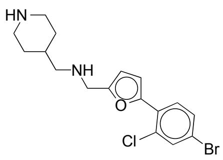	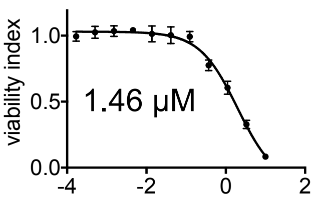 1.46 µM	Blood stage: 1.505 µM SMFA: 0.07 µM
F) MMV019881	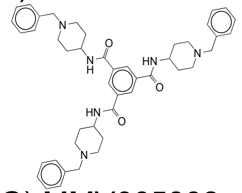	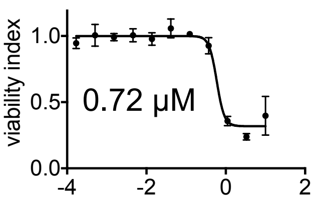 0.72 µM	Blood stage: 1.039 µM SMFA: 1.17 µM
G) MMV665882	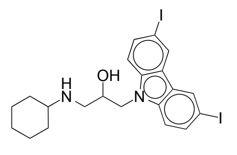	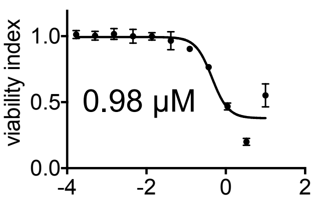 0.98 µM	Blood stage: 0.337 µM SMFA: not active
H) MMV667491	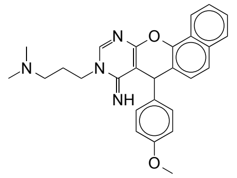	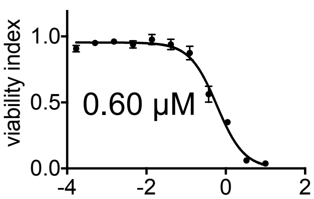 0.60 µM	Blood stage: 1.23 µM SMFA: 0.06 µM
I) MMV665980	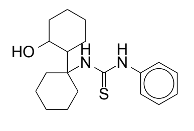	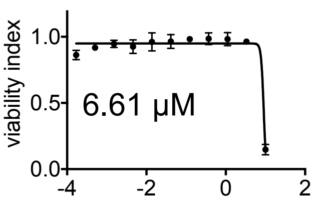 6.61 µM	Blood stage: 0.211 µM SMFA: 1.74 µM
J) MMV666125	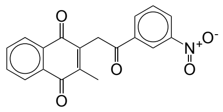	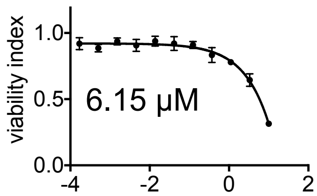 6.15 µM	Blood stage: 2.844 µM SMFA: 1.41 µM
K) MMV000442	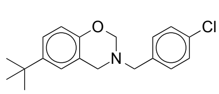	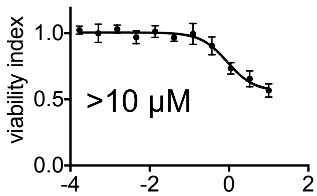 >10 µM	Blood stage: 0.255 µM SMFA: 0.59 µM
L) MMV000448	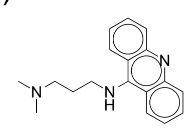	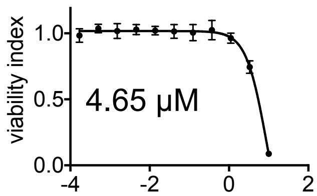 4.65 µM	Blood stage: 0.033 µM SMFA: 1.17 µM
M) MMV665971	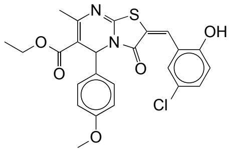	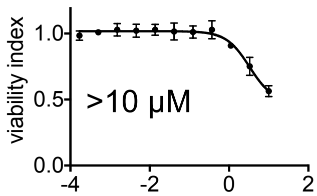 >10 µM	Blood stage: 0.746 µM SMFA: 3.24 µM
N) MMV396797	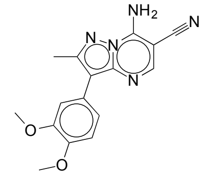	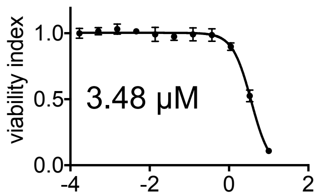 3.48 µM	Blood stage: 0.477 µM SMFA: 3.63 µM
O) MMV000248	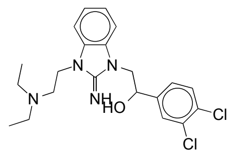	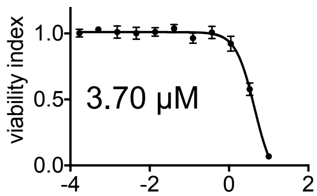 3.70 µM	Blood stage: 0.785 µM SMFA: 1.66 µM
P) MMV019266	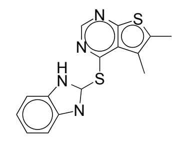	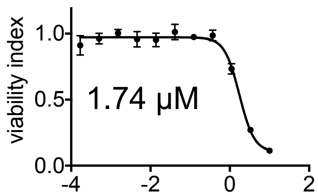 1.74 µM	Blood stage: 0.255 µM SMFA: 1.29 µM
Q) MMV011438	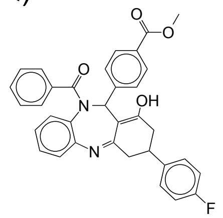	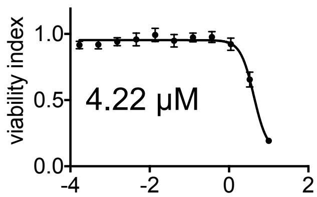 4.22 µM	Blood stage: 0.332 µM SMFA: 8.51 µM

Charts: y-axis "viability index" (0.0–1.0); x-axis "log compound [µM]" (−4 to 2).

Figure S3, related to Table 2 and Table S2. Identification of potential false negatives using a set of 17 control compounds of the MMV malaria box

Comparison of 17 control compounds (A-Q) derived from the MMV malaria box showing their chemical structure, their activity in dose response in 1536-well SaLSSA assay for stage V gametocytes with corresponding EC_{50} values (performed in duplicate) as well as EC_{50} values of asexual blood stage parasites (based on ChEMBL database) and EC_{50} values based on oocyst intensity in SMFA (performed at TropIQ, The Netherlands).

Table S1 (excel file), related to Table 1: MMV control compounds and known antimalarials

Table S2 (excel file), related to Table 2: MMV malaria box compounds

Table S3 (excel file), related to Figure 1 and 3: GNF malaria box compounds

Table S4 (excel file), related to Figure 1 and 3: Broad DOS compounds

Table S5

HEOS_COMPOUND_ID	ChEMBL_NTD_ID	SaLSSA (one-step protocol, 1536-well)						Ruecker et al., 2014				Sun et al., 2014	Duffy, Avery et al. 2013						Bowman et al., 2014
								Pf DGFA 1 µM male		Pf DGFA 1 µM female			NF54-pfs16-GFP early (I-III) gam			NF54-pfs16-GFPb late (IV-V) gam			
		EC50 stage I (µM)	Std stage I	EC50 stage III (µM)	Std stage III	EC50 stage V (µM)	Std stage V	% inhibition	SEM	% inhibition	SEM	IC50(µM)	5µM	0.5 µM	IC_{50} nM	5uM	0.5uM	IC_{50} nM	% Late stage Gametocyte Inhibition
MMV000442	SJ000043130	0.553	0.041	>10.000	0.000	>10.000	0.000	-12.20	16.93	6.43	6.79	0.332	98	10	100%	55	27	920	72
MMV665971	TCMDC-124112;SJ000154494	0.746	0.116	>10.000	0.000	>10.000	0.000	-29.23	17.74	0.52	-13.01	2.957	108	105	359	103	94	228	73
MMV011438	GNF-Pf-4836	1.120	0.116	2.428	0.620	4.225	0.609	-76.84	22.36	1.08	-31.41	5.258	103	27	80%	98	89	1130	100
MMV000248	TCMDC-124062;SJ000018645	1.058	0.026	3.074	0.533	3.700	0.173	-66.45	27.61	0.61	7.92	1.482	62	32	1444	89	19	1091	100
MMV666125	GNF-Pf-119	0.094	0.068	1.342	0.259	6.154	0.569	-2.58	11.62	-0.51	-17.41	0.833	60	48	1157	54	39	745	100
MMV019918	TCMDC-124617	1.264	0.145	0.576	0.069	1.463	0.296	74.09	11.71	-0.52	38.13	1.866	100	93	824	90	36	692	100
MMV019266	TCMDC-123835	0.935	0.214	1.372	0.210	1.743	0.206	30.77	9.27	-0.93	-10.93	11.77	91	90	1142	94	31	324	100
MMV396797	NA	5.435	0.470	2.094	0.424	3.477	0.175	-13.84	8.86	0.53	-16.78	6.619	23	-24		108	64	108 %	90
MMV667491	NA	0.980	0.069	0.667	0.101	0.596	0.085	83.58	12.98	-0.85	27.26	2.635	-23	76		115	108	1060	91
MMV019881	TCMDC-124568	3.048	3.278	8.408	1.577	0.721	0.141	57.05	10.02	-1.48	-6.87	2.957	82	20		32	0		76
MMV000448	GNF-Pf-2110;TCMDC-124266;SJ000044378	1.195	0.113	5.356	0.612	4.652	0.394	97.47	1.33	-2.21	24.60	1.866	101	85	1439	101	58	703	95
MMV665882	TCMDC-124074	0.180	0.036	1.477	0.136	0.984	0.022	-12.34	14.46	0.05	-48.03	2.957	95	61	252	98	85	63	92
MMV665941	SJ000140980	0.388	0.035	2.271	0.751	1.044	0.040	19.70	12.32	-0.63	44.59	5.899	-12	81	769	103	102	315	86
MMV665980	GNF-Pf-4228	>10.000	0.000	9.350	3.237	6.612	1.100	80.70	7.91	-0.97	73.75	11.77	72	3		91	34		27
MMV007116	GNF-Pf-4421	>10.000	0.000	>10.000	0.000	>10.000	0.000	65.80	8.34	-2.05	9.93	Inactive	43	-8		80	74	253	40
MMV665827	GNF-Pf-4731;TCMDC-124156	>10.000	0.000	>10.000	0.000	>10.000	0.000	92.37	3.62	-2.24	-15.24	Inactive	17	22	NA	93	91	337	41
MMV666021	GNF-Pf-3832	>10.000	0.000	>10.000	0.000	>10.000	0.000	30.43	9.73	-0.99	-20.39	Inactive	95	3	NA	71	102	603	58
MMV020492	TCMDC-125225	>10.000	0.000	>10.000	0.000	>10.000	0.000	35.61	9.10	-1.10	-12.33	Inactive	1	-11		-5	6		70

Table S5, related to Table 2 and Table S2.

Comparison of gametocytocidal activities of 18 control compounds of the MMV malaria box between the 1536-well SaLSSA assay for gametocytes stages I, III and V (experiments for EC_{50} values with standard deviation were performed in duplicates) and published data as referenced: Ruecker et al., 2014 (% inhibition in dual gamete formation assay (DGFA) at 1 μM; Sun et al., 2014 (IC50 values for gametocyte stages III-V, Alamar Blue Assay); Duffy and Avery, 2013 (% inhibition and IC50 values for early (stage I-III) and late (stage IV-V) gametocytes determined with gametocyte specific marker (pfs16-Luc-GFP) and the viability marker MitoTracker Red CM-H2XRos; Bowman et al., 2014 (% inhibition for late stage gametocytes with Alamar Blue Assay at 5 μM).

Supplemental Experimental Procedures

Parasite culture

Stage-specific gametocyte cultures of a clonal strain of NF54 were prepared as previously described (Fivelman et al., 2007) with some modifications: Asexual parasites were grown at 5% hematocrit in O+ human erythrocytes (Normal Blood Donor Service, The Scripps Research Institute, La Jolla, CA) in serum-containing complete media (RPMI 1640 (Life Technologies), gentamicin 0.05mg/ml (Life Technologies), hypoxanthine 0.014mg/ml (Sigma), HEPES 38.4mM (Sigma), sodium bicarbonate 0.2% (w/v) (Sigma), D-glucose 0.2% (w/v) (Sigma), sodium hydroxide 3.4mM (Sigma), 4.3% (v/v) heat inactivated human serum (O+, Interstate Blood bank) and 0.2% (w/v) AlbuMAX® II (Life Technologies)).

Parasites were cultured at 37ºC under low oxygen conditions (3% O_2, 5% CO_2, 92% N_2). During the initial induction phase (days ≤ -8 until day -4) parasitemia was adjusted between 0.5% and 3%. Ring-stage parasites underwent three rounds of synchronization (d-8, d-6, d-4) with 5% (wt/vol) D-Sorbitol (Sigma) while cultures were gradually expanded from T25 culture flasks (10ml total volume) to T225 culture flasks (100ml total volume). Trophozoite-stage parasites were shaken over night at 37º until d-1. The hematocrit was adjusted to 5% until day -4 and not lowered hereafter. On day -2 only 50% of the media was exchanged to provide additional stress to the high parasitemia of 7-10%. Complete media exchange was performed daily from day -1 onwards. For stages I-IV magnetically activated cell sorting (MACS) was performed on day 0 (rings) and sorbitol- synchronization on day 1. These two steps were omitted for stage V

gametocytes without loss of purity. All gametocytes were treated with 50mM NAG on days 0-9.

Stage specific gametocytemia was assessed morphologically by examining 1000-2000 red blood cells in Giemsa-stained thin blood smears (Carter and Miller, 1979, Talman et al., 2004). Only cultures with >75% stage specificity and no relevant contamination (<1%) with asexual parasites were included in the assays.

Two-step sexual stage assay (TSSA), 384 well format- all stages (high- throughput, serum-containing)

In the TSSA gametocyte stages I-V were assayed independently in compound-loaded black clear bottom 384 well plates (Greiner). Gametocyte cultures in serum-containing complete media at 0.50% gametocytemia and 1.25% hematocrit were dispensed into wells at a volume of 40µl. The complete media contained 50mM of N-Acetyl-D-Glucosamine for gametocyte stages I, II, III, and IV, and no N-Acetyl-D-Glucosamine for stage V. Plate lids were covered with breathable seals and placed on top of the assay plates. The plates were incubated at 37°C for 72 hours under low oxygen conditions. In a first step, 5µl from a 5µM solution of MitoTracker® Red CMXRos in screening media (RPMI 1640 (Life Technologies), gentamicin 0.05mg/ml (Life Technologies), hypoxanthine 0.014mg/ml (Sigma), HEPES 38.4mM (Sigma), sodium bicarbonate 0.2% (w/v) (Sigma), D-glucose 0.2% (w/v) (Sigma), sodium hydroxide 3.4mM (Sigma), 0.4% (w/v) AlbuMAX® II (Life Technologies)) was dispensed into the wells of the assay plates and incubated for 20 minutes at 37°C. Into a new 384 well imaging plate, 40µL of a 500nM solution of MitoTracker® Red CMXRos in serum-free screening media was dispensed per well. After mixing, 5µL from each well of the assay plate was transferred

into the imaging plate while resting on top of a 37°C heating pad. The imaging plate was sealed and rested for 30 minutes inside an Operetta high content imaging system before imaging.

One-step protocol (SaLSSA)-384 well plate format- all stages (high throughput, serum-free)

In the 384-well SaLSSA gametocytes stages I-V were assayed independently in compound-loaded black clear bottom 384 well plates (Greiner). Gametocyte cultures in serum-free screening media at 0.50% gametocytemia and 1.25% hematocrit were dispensed into wells at a volume of 40 µl. The screening media contained 50mM of N-Acetyl-D-Glucosamine for gametocyte stages I, II, III, and IV, and no N-Acetyl-D-Glucosamine for stage V. Plate lids were covered with breathable seals and placed on top of the assay plates. The plates were incubated at 37°C for 72 hours under low oxygen conditions. 10µl MitoTracker® Red CMXRos (2.5 µM) (Life Technologies) and saponin solution (0.13% w/v) (ACROS Organics, cat. no 419231000) in screening media were added. Assay plates containing media with N-Acetyl-D-Glucosamine were shaken for 2 minutes and incubated at 37°C for 90-120 minutes before analysis. Assay plates containing media without N-Acetyl-D-Glucosamine were incubated at 37°C for 60-90 minutes before analysis. The imaging plates were sealed, and rested for 30 minutes inside an Operetta high content imaging system before imaging.

One-step protocol (SaLSSA)-1536 well plate format- stage V (ultra-high-throughput capable, serum-free)

In the 1536-well SaLSSA stage V gametocytes were assayed in compound-loaded black clear bottom 1536-well plates (Greiner). Gametocyte cultures in screening media at

0.75% gametocytemia and 1.25% hematocrit were dispensed into wells at a volume of 10 µl using a MultiFlo (BioTek). A single culture of stage V gametocytes (100ml volume, 1.2% ±0.2% parasitemia) allowed us to screen 8-10 1536-well plates at a time reproducibly. Plate lids were covered with breathable seals and placed on top of the assay plates. The plates were incubated at 37°C for 72 hours under low oxygen conditions. 3 µl MitoTracker® Red CMXRos (2.5 µM) (Life Technologies, cat. no M-7512) and saponin solution (0.13% w/v) (ACROS Organics, cat. no 419231000) were added. Plates were incubated for 60-90 minutes at 37ºC. The plates were placed inside an Operetta high content imaging system for 30 minutes, and then imaged.

High-content imaging and analysis

Imaging of 384- or 1536-well plates was performed using a high content imaging system (Operetta R, Perkin Elmer). With a 10x wide field objective and an excitation⁄emission filter of 560-580⁄590-640 nm, three fields of view were imaged for compounds tested in 384 well plates, while one field of view was used for compounds tested in 1536 well plates. Both layouts could be imaged in ~45 minutes/plate. The gametocytocidal activity was calculated using a high-content imaging analysis software (Harmony, Perkin Elmer). The script was based on the following parameters: 1. Normalization of fluorescence intensity based on DMSO incubated wells; 2. Identification of fluorescent particles; 3. Inclusion of red fluorescent particles in the range of gametocytes (20-70μm^2); 4. Counting of red fluorescent particles in each well; 5. Normalization of particle count based on the particle number in wells incubated with the compound solvent DMSO: Calculation of the viability index was performed by dividing the particle number of each compound-treated well by the average particle number of the DMSO wells per plate.

Readouts generated a viability index close to 1 for compounds without gametocytocidal activity and close to 0 for compounds with complete gametocytocidal activity. Z-values were calculated using DMSO treated gametocytes as positive wells, and uninfected red blood cells as negative wells.

Statistical analysis:

IC_{50} were obtained using a custom curve-fitting model, and a standard logistic regression model was applied for curve fitting (Meister et al., 2011). Each experiment was performed in duplicate. For all other statistical analyses we used Prism 6 for Mac OS X (GraphPad Software, La Jolla, CA).

Compound Clustering:

13844 tested compounds (400 compounds from MMV-malaria box, 3558 compounds from GNF-malaria box and 9886 compounds from Broad Diversity-Oriented Synthesis Library) were organized into a tree hierarchy based on their scaffolds using the Scaffold Tree algorithm (Schuffenhauer et al., 2007) outlined below: (1) A hierarchy of ring scaffolds are enumerated from each molecule by removing terminal side chains first, and then removing the least characteristic ring components one at a time till a single ring is left. (2) All the ring scaffolds obtained from all molecules are then reassembled into a tree with increasing level of complexity, i.e., each node represents a unique ring scaffold and each level away from the root represents the increment of an additional ring to the parent scaffold. To facilitate our interpretation, the analysis here was limited to the first four levels of the scaffold hierarchy that correspond to 1- through 4-ring scaffolds. The scaffold hierarchy contains 322 distinct 1-ring scaffolds, 1080 2-ring scaffolds, 2416 3-ring scaffolds and 3439 4-ring scaffolds.

Each scaffold node was then assigned enrichment score reflecting the degree of overrepresentation of active compounds (stage V gametocyte inhibitors). More specifically, we calculated the accumulative hypergeometric p-value as probability of observing at least as many hits as we observed within each scaffold. The tree was then pruned, so that only scaffolds with p-values < 0.001 were retained. The final resultant tree in Figure 3 was rendered with Cytoscape (version 3.2.0). To focus on those nodes where the scaffold of a node could reasonably resemble the full structures of all associated compound members, the average Tanimoto similarity score between each scaffold node and its associated compounds were calculated based on ChemAxon topological fingerprints (ChemAxon, Kft.), and those tree nodes and leaves with at least 0.85 Tanimoto scores and with at least 3 hits are highlighted in colors in Figure 3.
